# 4-(3-Fluoro-4-nitro­phen­yl)morpholin-3-one

**DOI:** 10.1107/S1600536811019738

**Published:** 2011-05-28

**Authors:** Chang-Jiang Huang, Jiang Wu, Zhi-Qiang Cai, Jing Yuan

**Affiliations:** a Tianjin Key Laboratory of Molecular Design and Drug Discovery, Tianjin Institute of Pharmaceutical Research, Tianjin 300193, People’s Republic of China; b Tianjin Institute of Pharmaceutical Research, Tianjin 300193, People’s Republic of China

## Abstract

In the title compound, C_10_H_9_FN_2_O_4_, the dihedral angle between the benzene ring and the nitro group plane is 11.29 (3)°. The morpholinone ring adopts a twist-chair conformation. In the crystal, mol­ecules are linked by inter­molecular C—H⋯O hydrogen bonds into a chain along the *a*-axis direction.

## Related literature

The title compound is an inter­mediate in the preparation of derivatives of the factor Xa inhibitor rivaroxaban (systematic name (*S*)-5-chloro-*N*-{[2-oxo-3-[4-(3-oxomorpholin-4-yl)phen­yl]oxazolidin-5-yl]meth­yl}thio­phene-2-carboxamide). For the bioactivity and applications of rivaroxaban, see: Pinto *et al.* (2010 ▶); Haas (2008 ▶); Squizzato *et al.* (2009 ▶); Samama & Gerotziafas (2010 ▶); Van Huis *et al.* (2009 ▶). For the synthesis of other derivatives with morpholone, see: Van Huis *et al.* (2009 ▶); Zbinden *et al.* (2009 ▶).
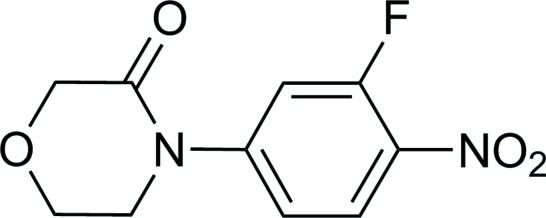


## Experimental

### 

#### Crystal data

C_10_H_9_FN_2_O_4_
*M* *_r_* = 240.19
Triclinic, 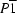

*a* = 6.6408 (7) Å
*b* = 7.3788 (10) Å
*c* = 10.8546 (14) Å
α = 73.30 (3)°
β = 75.39 (3)°
γ = 74.30 (3)°
*V* = 481.60 (14) Å^3^
*Z* = 2
Mo *K*α radiation
μ = 0.14 mm^−1^
*T* = 113 K
0.22 × 0.20 × 0.10 mm

#### Data collection

Rigaku Saturn CCD area-detector diffractometer
Absorption correction: ψ scan (*CrystalClear*; Rigaku/MSC, 2009 ▶) *T*_min_ = 0.970, *T*_max_ = 0.986
6470 measured reflections
2569 independent reflections
1734 reflections with *I* > 2σ(*I*)
*R*_int_ = 0.041

#### Refinement

*R*[*F* ^2^ > 2σ(*F* ^2^)] = 0.039
*wR*(*F* ^2^) = 0.104
*S* = 0.97
2569 reflections
154 parameters
H-atom parameters constrained
Δρ_max_ = 0.56 e Å^−3^
Δρ_min_ = −0.25 e Å^−3^

### 

Data collection: *RAPID-AUTO* (Rigaku, 1998 ▶); cell refinement: *RAPID-AUTO*; data reduction: *RAPID-AUTO*; program(s) used to solve structure: *SHELXS97* (Sheldrick, 2008 ▶); program(s) used to refine structure: *SHELXL97* (Sheldrick, 2008 ▶); molecular graphics: *SHELXTL* (Sheldrick, 2008 ▶); software used to prepare material for publication: *CrystalStructure* (Rigaku/MSC, 2009 ▶).

## Supplementary Material

Crystal structure: contains datablocks I, global. DOI: 10.1107/S1600536811019738/kp2328sup1.cif

Structure factors: contains datablocks I. DOI: 10.1107/S1600536811019738/kp2328Isup2.hkl

Supplementary material file. DOI: 10.1107/S1600536811019738/kp2328Isup3.cml

Additional supplementary materials:  crystallographic information; 3D view; checkCIF report

## Figures and Tables

**Table 1:** Hydrogen-bond geometry (Å, °)

*D*—H⋯*A*	*D*—H	H⋯*A*	*D*⋯*A*	*D*—H⋯*A*
C6—H6⋯O2^i^	0.95	2.39	3.2635 (16)	153
C2—H2*B*⋯O3^ii^	0.99	2.50	3.3244 (19)	140
C1—H1*B*⋯O4^iii^	0.99	2.57	3.515 (2)	161

Symmetry codes: (i) 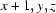; (ii) 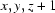; (iii) 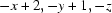.

**Fig. 1.**
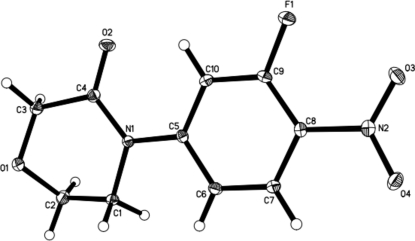
The structure of C10H9F1N2O4 with all non-H atom-labelling scheme and displacement ellipsoids drawn at the 50% probability level.

## supplementary crystallographic information

### Comment

Rivaroxaban is an oral, direct factor Xa inhibitor for the prevention and treatment of arterial and venous thrombosis (Pinto *et al.*, 2010; Haas, 2008; Squizzato *et al.*, 2009).

The title compound (Fig. 1) is important intermediate in the preparation of derivatives of Rivaroxaban. Some derivatives of Rivaroxaban have been reported for having high affinity for human FXa (Squizzato *et al.*, 2009; Samama *et al.*, 2010; Van Huis *et al.*, 2009). Herein, the synthesis and the crystal structure of the title compound are reported.

In the title compound, C_10_H_9_F_1_N_2_O_4_, the dihedral angle between benzene ring and the plane of nitro group is 11.29 (3)°. The morpholone ring adopts a twist-chair conformation. In the crystal packing molecules are linked by intermolecular C—H···O hydrogen bonds into a chain (Table 1).

### Experimental

Potassium carbonate (6.73 g, 0.0488 mol) was added to a suspension of 2-(2-chloroethoxy)-*N*-(3-fluoro-4-nitrophenyl)acetamide (9.00 g, 0.0325 mol) in acetonitrile (200 mL).The reaction mixture was stirred at 385 K for 5 h. The mixture was evaporated in vacuo. Water was added.The reaction mixture was filtered, washed with water, and dried to obtain yellow solid (7.19 g).Colourless single crystals suitable for X-ray diffraction were obtained by recrystallisation from ethanol and ethyl acetate.

### Refinement

All H atoms were geometrically positioned (C—H 0.95–0.99 Å) and treated as riding, with *U*_iso_(H) = 1.2Ueq(C).

### Crystal data

**Table:**

C_10_H_9_FN_2_O_4_	*Z* = 2
*M**_r_* = 240.19	*F*(000) = 248
Triclinic, *P*1	*D*_x_ = 1.656 Mg m^−^^3^
Hall symbol: -P 1	Mo *K*α radiation, λ = 0.71073 Å
*a* = 6.6408 (7) Å	Cell parameters from 1909 reflections
*b* = 7.3788 (10) Å	θ = 2.0–31.1°
*c* = 10.8546 (14) Å	µ = 0.14 mm^−^^1^
α = 73.30 (3)°	*T* = 113 K
β = 75.39 (3)°	Prism, colourless
γ = 74.30 (3)°	0.22 × 0.20 × 0.10 mm
*V* = 481.60 (14) Å^3^	

### Data collection

**Table:**

Rigaku Saturn CCD area-detector diffractometer	2569 independent reflections
Radiation source: rotating anode	1734 reflections with *I* > 2σ(*I*)
multilayer	*R*_int_ = 0.041
Detector resolution: 14.63 pixels mm^-1^	θ_max_ = 29.1°, θ_min_ = 2.0°
ω and φ scans	*h* = −9→9
Absorption correction: ψ scan (*CrystalClear*; Rigaku/MSC, 2009)	*k* = −10→9
*T*_min_ = 0.970, *T*_max_ = 0.986	*l* = −14→14
6470 measured reflections	

### Refinement

**Table:**

Refinement on *F*^2^	Primary atom site location: structure-invariant direct methods
Least-squares matrix: full	Secondary atom site location: difference Fourier map
*R*[*F*^2^ > 2σ(*F*^2^)] = 0.039	Hydrogen site location: inferred from neighbouring sites
*wR*(*F*^2^) = 0.104	H-atom parameters constrained
*S* = 0.97	*w* = 1/[σ^2^(*F*_o_^2^) + (0.0502*P*)^2^] where *P* = (*F*_o_^2^ + 2*F*_c_^2^)/3
2569 reflections	(Δ/σ)_max_ < 0.001
154 parameters	Δρ_max_ = 0.56 e Å^−^^3^
0 restraints	Δρ_min_ = −0.25 e Å^−^^3^

### Special details

**Table:**

Geometry. All e.s.d.'s (except the e.s.d. in the dihedral angle between two l.s. planes) are estimated using the full covariance matrix. The cell e.s.d.'s are taken into account individually in the estimation of e.s.d.'s in distances, angles and torsion angles; correlations between e.s.d.'s in cell parameters are only used when they are defined by crystal symmetry. An approximate (isotropic) treatment of cell e.s.d.'s is used for estimating e.s.d.'s involving l.s. planes.
Refinement. Refinement of *F*^2^ against ALL reflections. The weighted *R*-factor *wR* and goodness of fit *S* are based on *F*^2^, conventional *R*-factors *R* are based on *F*, with *F* set to zero for negative *F*^2^. The threshold expression of *F*^2^ > σ(*F*^2^) is used only for calculating *R*-factors(gt) *etc*. and is not relevant to the choice of reflections for refinement. *R*-factors based on *F*^2^ are statistically about twice as large as those based on *F*, and *R*- factors based on ALL data will be even larger.

### Fractional atomic coordinates and isotropic or equivalent isotropic displacement parameters (Å^2^)

**Table:**

	*x*	*y*	*z*	*U*_iso_*/*U*_eq_	
F1	0.38071 (13)	0.73231 (13)	−0.24062 (8)	0.0292 (2)	
O1	0.23003 (15)	0.94560 (12)	0.40672 (8)	0.0192 (2)	
O2	0.04787 (15)	0.82852 (15)	0.16307 (9)	0.0262 (2)	
O3	0.74637 (16)	0.59970 (13)	−0.37865 (9)	0.0241 (2)	
O4	1.03428 (15)	0.62270 (13)	−0.32888 (9)	0.0234 (2)	
N1	0.39251 (16)	0.79057 (14)	0.18692 (10)	0.0139 (2)	
N2	0.84043 (18)	0.63168 (15)	−0.30564 (10)	0.0165 (2)	
C1	0.5325 (2)	0.77071 (18)	0.27977 (12)	0.0161 (3)	
H1A	0.6152	0.8739	0.2456	0.019*	
H1B	0.6347	0.6443	0.2855	0.019*	
C2	0.4068 (2)	0.78419 (19)	0.41571 (12)	0.0201 (3)	
H2A	0.3550	0.6634	0.4599	0.024*	
H2B	0.5001	0.7996	0.4685	0.024*	
C3	0.0821 (2)	0.90024 (19)	0.35316 (12)	0.0183 (3)	
H3A	−0.0349	1.0155	0.3382	0.022*	
H3B	0.0196	0.7956	0.4183	0.022*	
C4	0.1736 (2)	0.83638 (18)	0.22515 (12)	0.0164 (3)	
C5	0.4968 (2)	0.75696 (16)	0.06139 (12)	0.0132 (3)	
C6	0.7205 (2)	0.71941 (17)	0.03016 (12)	0.0160 (3)	
H6	0.7988	0.7192	0.0924	0.019*	
C7	0.8283 (2)	0.68282 (17)	−0.09000 (12)	0.0161 (3)	
H7	0.9795	0.6591	−0.1095	0.019*	
C8	0.7182 (2)	0.68025 (17)	−0.18264 (12)	0.0143 (3)	
C9	0.4967 (2)	0.72366 (18)	−0.15317 (12)	0.0158 (3)	
C10	0.3858 (2)	0.76283 (17)	−0.03458 (12)	0.0160 (3)	
H10	0.2345	0.7938	−0.0178	0.019*	

### Atomic displacement parameters (Å^2^)

**Table:**

	*U*^11^	*U*^22^	*U*^33^	*U*^12^	*U*^13^	*U*^23^
F1	0.0201 (5)	0.0495 (5)	0.0226 (4)	−0.0038 (4)	−0.0079 (4)	−0.0158 (4)
O1	0.0176 (5)	0.0226 (5)	0.0198 (5)	−0.0014 (4)	−0.0046 (4)	−0.0102 (4)
O2	0.0134 (5)	0.0476 (6)	0.0235 (5)	−0.0093 (5)	−0.0009 (4)	−0.0173 (5)
O3	0.0289 (6)	0.0285 (5)	0.0192 (5)	−0.0068 (4)	−0.0067 (4)	−0.0099 (4)
O4	0.0162 (5)	0.0297 (5)	0.0214 (5)	−0.0027 (4)	0.0012 (4)	−0.0078 (4)
N1	0.0125 (5)	0.0173 (5)	0.0125 (5)	−0.0031 (4)	−0.0020 (4)	−0.0049 (4)
N2	0.0192 (6)	0.0143 (5)	0.0148 (5)	−0.0029 (5)	−0.0020 (5)	−0.0028 (4)
C1	0.0135 (6)	0.0200 (7)	0.0159 (6)	−0.0013 (5)	−0.0055 (5)	−0.0056 (5)
C2	0.0196 (7)	0.0234 (7)	0.0158 (6)	0.0000 (6)	−0.0051 (5)	−0.0053 (5)
C3	0.0162 (7)	0.0218 (7)	0.0173 (6)	−0.0031 (5)	−0.0026 (5)	−0.0063 (5)
C4	0.0147 (7)	0.0177 (7)	0.0163 (6)	−0.0045 (5)	−0.0007 (5)	−0.0044 (5)
C5	0.0153 (6)	0.0117 (6)	0.0129 (6)	−0.0041 (5)	−0.0021 (5)	−0.0029 (5)
C6	0.0154 (7)	0.0182 (7)	0.0162 (6)	−0.0045 (5)	−0.0048 (5)	−0.0043 (5)
C7	0.0133 (6)	0.0181 (7)	0.0175 (6)	−0.0041 (5)	−0.0027 (5)	−0.0047 (5)
C8	0.0161 (7)	0.0134 (6)	0.0132 (6)	−0.0033 (5)	−0.0014 (5)	−0.0036 (5)
C9	0.0166 (7)	0.0180 (7)	0.0153 (6)	−0.0042 (5)	−0.0071 (5)	−0.0036 (5)
C10	0.0111 (6)	0.0188 (7)	0.0183 (6)	−0.0018 (5)	−0.0035 (5)	−0.0051 (5)

### Geometric parameters (Å, °)

**Table:**

F1—C9	1.3438 (13)	C2—H2B	0.9900
O1—C3	1.4118 (14)	C3—C4	1.5223 (18)
O1—C2	1.4286 (16)	C3—H3A	0.9900
O2—C4	1.2193 (14)	C3—H3B	0.9900
O3—N2	1.2292 (13)	C5—C10	1.4050 (16)
O4—N2	1.2349 (13)	C5—C6	1.4061 (18)
N1—C4	1.3823 (16)	C6—C7	1.3831 (17)
N1—C5	1.4223 (16)	C6—H6	0.9500
N1—C1	1.4885 (15)	C7—C8	1.3910 (16)
N2—C8	1.4630 (16)	C7—H7	0.9500
C1—C2	1.5171 (18)	C8—C9	1.3912 (18)
C1—H1A	0.9900	C9—C10	1.3795 (18)
C1—H1B	0.9900	C10—H10	0.9500
C2—H2A	0.9900		
			
C3—O1—C2	107.55 (9)	C4—C3—H3B	108.5
C4—N1—C5	123.48 (11)	H3A—C3—H3B	107.5
C4—N1—C1	120.11 (10)	O2—C4—N1	124.24 (12)
C5—N1—C1	116.39 (10)	O2—C4—C3	117.50 (12)
O3—N2—O4	123.82 (11)	N1—C4—C3	118.25 (11)
O3—N2—C8	118.74 (11)	C10—C5—C6	118.08 (11)
O4—N2—C8	117.43 (10)	C10—C5—N1	122.88 (11)
N1—C1—C2	112.26 (10)	C6—C5—N1	119.03 (11)
N1—C1—H1A	109.2	C7—C6—C5	120.88 (12)
C2—C1—H1A	109.2	C7—C6—H6	119.6
N1—C1—H1B	109.2	C5—C6—H6	119.6
C2—C1—H1B	109.2	C6—C7—C8	120.88 (12)
H1A—C1—H1B	107.9	C6—C7—H7	119.6
O1—C2—C1	109.95 (10)	C8—C7—H7	119.6
O1—C2—H2A	109.7	C7—C8—C9	118.10 (12)
C1—C2—H2A	109.7	C7—C8—N2	118.60 (11)
O1—C2—H2B	109.7	C9—C8—N2	123.31 (11)
C1—C2—H2B	109.7	F1—C9—C10	116.88 (12)
H2A—C2—H2B	108.2	F1—C9—C8	121.12 (12)
O1—C3—C4	114.95 (11)	C10—C9—C8	121.99 (12)
O1—C3—H3A	108.5	C9—C10—C5	119.97 (12)
C4—C3—H3A	108.5	C9—C10—H10	120.0
O1—C3—H3B	108.5	C5—C10—H10	120.0
			
C4—N1—C1—C2	6.24 (15)	N1—C5—C6—C7	178.93 (10)
C5—N1—C1—C2	−172.77 (10)	C5—C6—C7—C8	−0.66 (19)
C3—O1—C2—C1	70.39 (13)	C6—C7—C8—C9	2.70 (19)
N1—C1—C2—O1	−46.84 (14)	C6—C7—C8—N2	−177.12 (11)
C2—O1—C3—C4	−52.83 (13)	O3—N2—C8—C7	168.82 (11)
C5—N1—C4—O2	9.8 (2)	O4—N2—C8—C7	−10.19 (16)
C1—N1—C4—O2	−169.11 (11)	O3—N2—C8—C9	−10.99 (18)
C5—N1—C4—C3	−170.40 (11)	O4—N2—C8—C9	170.00 (11)
C1—N1—C4—C3	10.66 (17)	C7—C8—C9—F1	176.97 (10)
O1—C3—C4—O2	−167.37 (11)	N2—C8—C9—F1	−3.22 (19)
O1—C3—C4—N1	12.84 (16)	C7—C8—C9—C10	−1.95 (19)
C4—N1—C5—C10	−1.57 (18)	N2—C8—C9—C10	177.87 (11)
C1—N1—C5—C10	177.41 (11)	F1—C9—C10—C5	−179.83 (10)
C4—N1—C5—C6	177.30 (11)	C8—C9—C10—C5	−0.87 (19)
C1—N1—C5—C6	−3.73 (16)	C6—C5—C10—C9	2.90 (18)
C10—C5—C6—C7	−2.16 (18)	N1—C5—C10—C9	−178.23 (11)

### Hydrogen-bond geometry (Å, °)

**Table:**

*D*—H···*A*	*D*—H	H···*A*	*D*···*A*	*D*—H···*A*
C6—H6···O2^i^	0.95	2.39	3.2635 (16)	153
C2—H2B···O3^ii^	0.99	2.50	3.3244 (19)	140
C1—H1B···O4^iii^	0.99	2.57	3.515 (2)	161

Symmetry codes: (i) *x*+1, *y*, *z*; (ii) *x*, *y*, *z*+1; (iii) −*x*+2, −*y*+1, −*z*.
